# Enteric Glia Play a Critical Role in Promoting the Development of Colorectal Cancer

**DOI:** 10.3389/fonc.2020.595892

**Published:** 2020-11-13

**Authors:** Robert Yuan, Nupur Bhattacharya, Justin A. Kenkel, Jeanne Shen, Michael A. DiMaio, Sreya Bagchi, Tyler R. Prestwood, Aida Habtezion, Edgar G. Engleman

**Affiliations:** ^1^Department of Pathology, Stanford University School of Medicine (Blood Center), Palo Alto, CA, United States; ^2^Department of Pathology, Stanford University School of Medicine, Stanford, CA, United States; ^3^Department of Pathology, Marin Medical Laboratories, Novato, CA, United States; ^4^Institute for Stem Cell Biology and Regenerative Medicine, Stanford University School of Medicine, Stanford, CA, United States; ^5^Division of Gastroenterology and Hepatology, School of Medicine, Stanford University, Stanford, CA, United States

**Keywords:** enteric glia, colorectal cancer, azoxymethane/dextran sodium sulfate, Apc^Min/+^, enteric nervous system

## Abstract

Enteric glia are a distinct population of peripheral glial cells in the enteric nervous system that regulate intestinal homeostasis, epithelial barrier integrity, and gut defense. Given these unique attributes, we investigated the impact of enteric glia depletion on tumor development in azoxymethane/dextran sodium sulfate (AOM/DSS)-treated mice, a classical model of colorectal cancer (CRC). Depleting GFAP^+^ enteric glia resulted in a profoundly reduced tumor burden in AOM/DSS mice and additionally reduced adenomas in the *Apc^Min^*^/+^ mouse model of familial adenomatous polyposis, suggesting a tumor-promoting role for these cells at an early premalignant stage. This was confirmed in further studies of AOM/DSS mice, as enteric glia depletion did not affect the properties of established malignant tumors but did result in a marked reduction in the development of precancerous dysplastic lesions. Surprisingly, the protective effect of enteric glia depletion was not dependent on modulation of anti-tumor immunity or intestinal inflammation. These findings reveal that GFAP^+^ enteric glia play a critical pro-tumorigenic role during early CRC development and identify these cells as a potential target for CRC prevention.

## Introduction

The enteric nervous system is critical for regulating gut homeostatic processes (1, 2) and is comprised of two main cell types: enteric neurons and enteric glia. While the enteric neurons regulate contractility of the gut (3, 4), the enteric glia—a transcriptionally unique population of peripheral glia (5)—function to support the neurons and modulate neuronal activity (1, 6, 7). However, the role of these cells extends beyond just neuron support, as studies have implicated enteric glia in regulating epithelial barrier integrity (8–11), epithelial cell proliferation and differentiation (12–14), and gut defense (15–17). Despite these important functions, however, their impact on the development of colorectal cancer (CRC) has not been well investigated. Nevertheless, a number of observations suggest that these cells may be involved in modulating tumor development, as they are implicated in the origin of gastrinomas (18), they can produce inflammatory molecules (19–21), and their tissue organization is disrupted in human neoplasias (13, 22). Moreover, megacolon, which can develop following ablation of the ganglia in the gut, appears to protect against carcinogenesis (23, 24). Enteric glia have additionally been demonstrated to engage in a crosstalk with colon cancer stem cells *in vitro* that promotes cancer cell growth and stemness (25). However, investigations of their contribution to CRC development using *in vivo* tumor models have yet to be carried out.

Here, using the azoxymethane/dextran sodium sulfate (AOM/DSS) and Apc^Min/+^ mouse intestinal tumor models, we demonstrate that enteric glia contribute to the earliest stages of CRC development, and that their pro-tumorigenic function is not dependent on modulation of anti-tumor immunity or intestinal inflammation. These findings suggest that strategies targeting these cells could prove useful for CRC prevention.

## Materials and Methods

### Patient Specimens

De-identified CRC tumors and surrounding tissue were obtained under protocols approved by the Stanford Institutional Review Board. Tissues were formalin fixed and paraffin embedded for histology.

### Mice

Wild-type C57BL/6J mice and breeding pairs of *Apc^Min^*^/+^, *Gfap*-tk (thymidine kinase), *Gfap*-Cre, *Plp1*-CreER^T^, inducible diphtheria toxin receptor (iDTR), *Rag2*^-/-^ γc^-/-^, *Myd88*^fl/fl^, *Ifngr1*^fl/fl^, *Rela*^fl/fl^, and *Stat3*^fl/fl^ mice were purchased from The Jackson Laboratory. All mice were housed in an American Association for the Accreditation of Laboratory Animal Care-accredited animal facility and maintained in specific pathogen-free conditions on wood-chip bedding and fed standard rodent chow *ad libitum* unless otherwise stated. Littermates were used for controls whenever possible; if different litters had to be combined, mice were age matched and non-littermates were co-housed to eliminate effects of the microbiome. Mice of both sexes were utilized in the experiments performed in this study, and experiments were carried out during the light cycle. All animal care and experimentation were approved by the Stanford University Institutional Animal Care and Use Committee.

### DSS and AOM/DSS Mouse Models

The dextran sodium sulfate (DSS) and azoxymethane/dextran sodium sulfate (AOM/DSS) mouse models of colitis and CRC, respectively, were established using a protocol adapted from Wirtz et al. (26), with slight modifications. In brief, for inducing acute colitis, nine- to eleven-week-old mice were injected intraperitoneally (i.p.) with 10 mg/kg AOM (Sigma-Aldrich) and given 3% DSS salt (36,000–50,000 Da; MP Biomedicals) in drinking water for five days, followed by a return to normal drinking water. Disease activity index was calculated by summing the scores for body weight loss (0 = none, 1 = 0–5%, 2 = 5–10%, 3 = 10–15%, 4 = >15%), stool consistency (0 = normal, 2 = loose pellet, 4 = diarrhea), fecal blood (0 = none, 2 = positive), and rectal bleeding (0 = none, 2 = positive). Colon length, inflammatory infiltrate, and histopathology were analyzed ten days post DSS administration. For establishing CRC, nine- to eleven-week-old mice were given 10 mg/kg AOM i.p. followed by three cycles of 3% DSS (one cycle consists of five days on DSS followed by sixteen days on normal water). Additional DSS cycles were continued if further tumor growth was desired. Controls for the experiments with the *Gfap*-Cre conditional knockout mice (*Rela*^fl/fl^, *Ifngr1*^fl/fl^, *Myd88*^fl/fl^, *Stat3*^fl/fl^) were non-Cre flox/flox littermates. Tumor burden was calculated by summing the sizes of the individual tumors. For assessing dysplasia, colons were harvested at six weeks post induction.

### Drug Treatment and BrdU Injections

For depletion of enteric glia in *Gfap*-tk mice, 25 mg/kg ganciclovir (Par Pharmaceutical) was injected i.p. into mice twice a week starting at either the time of AOM/DSS induction, three weeks after induction, or nine weeks after induction until the experimental endpoint. For depletion of enteric glia in *Apc^Min^*^/+^ *Gfap*-tk mice, 25 mg/kg ganciclovir was injected i.p. twice a week starting at six weeks of age and was continued for nine weeks until euthanization. For tracking cell proliferation, 1 mg of bromodeoxyuridine (BrdU) was injected i.p. 18 h before harvest.

### Intracolonic Injections

For enteric glia depletion, nine- to eleven-week-old *Gfap*-Cre iDTR or *Plp1*-CreER^T^ iDTR were injected with 20 ng of diphtheria toxin (DT) (Sigma-Aldrich) in 20 µL PBS mixed with Evan’s blue dye in the distal colon. The injections were guided by a Karl Storz colonoscopy system and performed with a custom-made 30-gauge needle end attached to a syringe *via* capillary tubing. Injections were performed four mm from the end of the rectum and were repeated once two days later. For *Plp1*-CreER^T^ iDTR mice, 1.5 mg 4-hydroxytamoxifen (Sigma-Aldrich), dissolved in 100% ethanol and emulsified in corn oil (Sigma Aldrich), was injected i.p. three and seven days prior to the start of the DT injections (two total injections of 4-hydroxytamoxifen). Control mice in these experiments were either *Gfap*-Cre iDTR and iDTR-only mice injected with PBS or iDTR-only mice injected with DT, depending on the Cre transgene distribution in the cohort; no difference in tumor burden results was observed between the two control groups. Control groups for the *Plp1*-CreER^T^ experiments were arranged in the same manner. Injected mice were then induced with AOM/DSS one week or three weeks after the initial injection for the tumor and colitis experiments, respectively. For final analyses, the distal-most two centimeters of the colon were collected to ensure analysis in an area of robust depletion.

### CD8^+^ T Cell and NK Cell Depletion

To deplete CD8^+^ T cells or natural killer (NK) cells in AOM/DSS mice, 250 7µg of rat anti-mouse CD8α antibody (clone YTS169.4, BioXCell) or 50 µg of mouse anti-mouse NK1.1 (clone PK136, BioXCell) was injected i.p. into wild-type or *Gfap*-tk mice twice a week from the start of induction. Control mice received the same amount of isotype control antibody: clone LTF2 and clone C1.18.4 for anti-CD8α and anti-NK1.1, respectively.

### Histology and Neoplasia and Colitis Grading

Formalin-fixed and paraffin-embedded colon tissues were sectioned at 5 µm and stained with hematoxylin and eosin. Neoplasia assessment and colitis grading were carried out by experienced gastrointestinal pathologists blinded to the sample groups. For neoplasia enumeration and grading, Swiss rolls of colons were step-sectioned every 400 µm until the tissue was exhausted to facilitate a complete evaluation of the total number of lesions in the colon. For colitis grading, the scheme from Seamons et al. (27) was used.

### Immunofluorescence Staining

Colon samples from mice were embedded in optimal cutting temperature compound (OCT, Tissue-Tek) and sectioned at 8–10 µm. Sections were post-fixed in 2% paraformaldehyde and blocked with 5% goat serum (Dako) or 5% donkey serum (Abcam) in 1% bovine serum albumin and subsequently stained with primary antibody. For staining SOX10 or HuC/D, sections were incubated in −20°C absolute methanol for 10 minutes prior to blocking. For staining BrdU, sections underwent antigen retrieval with the Diva Decloaker (Biocare Medical) and were incubated with 1 N HCl for one hour at room temperature before blocking. The primary antibodies used in this work were rat anti-GFAP (clone 2.2B10, Life Technologies, 1:300), mouse anti-HuC/D (Invitrogen, 1:200), goat anti-SOX10 (Santa Cruz Biotechnology, 1:200), rabbit anti-β-III-tubulin (Abcam, 1:200), and rabbit anti-pH2A.X (Cell Signaling, 1:200)), rat anti-BrdU (clone BU1/75 (ICR1), Abcam, 1:200), rat anti-EpCAM biotin (clone G8.8, Biolegend, 1:200), and rat anti-CD31 (clone MEC13.3, Biolegend, 1:200). Isotype stains were performed using goat, mouse, rat, and rabbit total IgG (Jackson ImmunoResearch) at identical concentrations as the respective primary antibody. The secondary stains used were streptavidin Alexa Fluor 488 (Life Technologies, 1:200), goat anti-rat Alexa Fluor 488 (Life Technologies, 1:200), goat anti-rat Alexa Fluor 594 (Life Technologies, 1:200), goat anti-rabbit Alexa Fluor 488 (Life Technologies, 1:200), goat anti-rabbit Alexa Fluor 594 (Life Technologies, 1:200), goat anti-mouse Alexa Fluor 594 (Life Technologies, 1:200), donkey anti-rabbit Alexa Fluor 488 (Life Technologies, 1:200), and donkey anti-goat Alexa Fluor 594 (Life Technologies, 1:200). DAPI was used as a nuclear stain; and terminal deoxynucleotidyl transferase dUTP nick end labeling (TUNEL) staining on mouse colonic sections was performed according to the manufacturer’s instructions (Roche Applied Sciences). Human colonic tissue specimens were acquired from formalin-fixed paraffin-embedded blocks as 5-µm-thick sections. Antigen retrieval was performed using the Diva Decloaker, then sections were blocked in 5% goat serum in 1% bovine serum albumin. The sections were subsequently stained with rabbit anti-S100β (clone EP1576Y, Abcam, 1:200). Goat anti-rabbit Alexa Fluor 594 (Life Technologies, 1:200) was used as a secondary stain. DAPI was used as a nuclear stain. Images were collected using a Zeiss 700 confocal laser scanning microscope or a Keyence BZ-X810 widefield fluorescent microscope and analyzed using ImageJ. For immunofluorescent image quantification, multiple fields of view were taken for each section and were analyzed in a blinded fashion. For quantification of S100β and GFAP, the mucosa was demarcated and analyzed for total area of S100β or GFAP staining within the total mucosal area.

### ELISA

Distal colonic lysates for AOM/DSS mice or small intestinal lysates for *Apc^Min^*^/+^ mice were prepared using RIPA buffer and assessed for protein concentration using the Micro BCA kit (Pierce). For *Gfap*-tk mice, the distal half of the colon was harvested for lysates while, for *Gfap*-Cre iDTR mice, the distal-most two centimeters were harvested. Lysate protein concentrations were normalized to 900 µg/mL and analyzed undiluted using a GFAP ELISA kit (R&D Systems) or a β-III-tubulin ELISA kit (MyBioSource).

### Flow Cytometry

To assess CD8^+^ T cell and NK cell depletion, blood was collected, and red blood cells were lysed. The remaining cells were then blocked with anti-mouse CD16/32 (Biolegend) and stained with antibodies against CD45 (clone 30-F11, Biolegend), CD3ϵ (clone 145-2C11, Biolegend), CD8α (clone 53-6.7, Biolegend), CD4 (clone GK1.5, Biolegend), CD49b (clone DX5, Biolegend), NK1.1 (clone PK136, Biolegend), CD11b (clone M1/70, Biolegend), and MHC II (clone M5/114.15.2, Biolegend). For analysis of immune cells in colitis, distal colon tissue was harvested from colitic mice and cut into 0.5 cm pieces. Intestinal pieces were then stirred in Hank’s balanced salt solution (HBSS) with dithiothreitol (Invitrogen) to remove mucus and subsequently digested with Liberase TL (Roche Applied Sciences) and DNase I (Sigma-Aldrich) in RPMI with 2% fetal bovine serum (FBS) for 45 minutes at 37°C. The remaining tissue was then mashed and filtered through a 100 µm filter to obtain a single-cell suspension. Cells were resuspended in FACS buffer containing EDTA and 2% FBS in HBSS. Cells were then blocked with anti-mouse CD16/32 (Biolegend) and stained with antibodies against CD45 (clone 30-F11, Biolegend), MHC II (clone M5/114.15.2, Biolegend), CD11b (clone M1/70, Biolegend), Ly6G (clone 1A8, Biolegend), Ly6C (clone HK1.4, Biolegend), F4/80 (clone BM8, Biolegend), and CD11c (clone N418, Biolegend). Live/Dead Aqua (Life Technologies) was used as a viability stain, and absolute cell counts were obtained using AccuCount fluorescent particles (Spherotech). Flow cytometric data acquisition was performed on LSRII and LSRFortessa flow cytometers (BD Biosciences) and the data analyzed with FlowJo (Treestar).

### Statistics

Experimental data were analyzed with the Mann-Whitney U-test using Prism (GraphPad Software), unless otherwise stated. All statistical tests performed are two sided. Results are presented as mean ± SEM. *P*<0.05 = *; *P*<0.01 = **; *P*<0.001 = ***; *P*<0.0001 = ****.

## Results

### Depletion of GFAP^+^ Enteric Glia Reduces Tumor Burden in AOM/DSS Mice

To establish CRC in mice, we used the azoxymethane/dextran sodium sulfate (AOM/DSS) model of colon cancer, which is widely used for studying CRC as it mimics human CRC both developmentally and histologically (28). Tumors in this model form primarily in the distal colon and are induced *via* a single injection of the colonotropic mutagen AOM followed by repeated oral administration of the inflammatory agent DSS. Dysplastic lesions appear approximately three to six weeks after induction, while overt tumors develop after approximately seven weeks and are primarily localized to the distal colon.

**Figure 1:**
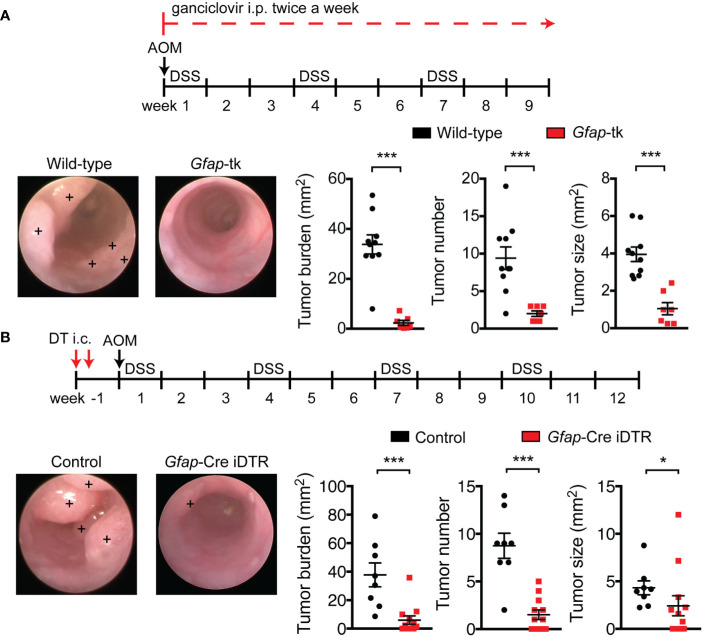
Enteric glia depletion reduces tumor burden in AOM/DSS mice. **(A, B)** Analysis of tumors in wild-type and *Gfap*-tk AOM/DSS mice treated with ganciclovir (n = 7–10 mice per group; pooled from two independent experiments) **(A)** and of non-depleted control AOM/DSS mice and enteric-glia-depleted *Gfap*-Cre iDTR AOM/DSS mice (n = 8–12 mice per group; pooled from two independent experiments) **(B)**. Representative colonoscopy images are shown, with tumors marked by a +. Data are presented as mean ± SEM. *P* < 0.05 = *; *P* < 0.001 = ***, Mann-Whitney U test.

To investigate whether enteric glia contribute to CRC, we utilized *Gfap*-tk transgenic mice, in which glial fibrillary acidic protein (GFAP)-expressing cells are rendered sensitive to ganciclovir-mediated cell depletion due to expression of a viral thymidine kinase (tk). After nine weeks of ganciclovir treatment, GFAP^+^ enteric glia, but not β-III-tubulin^+^ enteric neurons, were well depleted from the colons of AOM/DSS *Gfap*-tk mice (**Figures S1A, B**). As “bystander effects” and off-target cell death have been reported in tk-depletion systems and specifically in the *Gfap*-tk mice (29, 30), we sought to further investigate the selectivity of the cell depletion. When we stained colonic sections for pH2A.X, a marker of cell death, we observed that pH2A.X was selectively stained in cells expressing SOX10 and lacking HuC/D (**Figure S1C**), which are transcription factors identifying enteric glia and enteric neurons, respectively. This indicates that cell death occurred selectively in enteric glial cells with no bystander effects on adjoining neurons. The integrity of the neuronal network was additionally assessed by measuring the total levels of β-III-tubulin in distal colonic lysates from *Gfap*-tk and wild-type mice treated with ganciclovir. Colons from enteric-glia-depleted *Gfap*-tk mice compared to non-depleted wild-type mice demonstrated equivalent levels of β-III-tubulin (**Figure S1D**), indicating enteric glia depletion without concomitant depletion of enteric neurons. Damage to the small intestinal epithelium following ganciclovir administration has also been previously reported in the *Gfap*-tk system (11, 30), but no such epithelial damage was observed in our studies (**Figure S1E**), likely due to the reduced ganciclovir dosage used. Given the selective depletion of GFAP^+^ enteric glial cells achieved with this regimen, we assessed overall tumor burden in ganciclovir-treated *Gfap*-tk and wild-type mice nine weeks after AOM/DSS induction and discovered that the tumor burden of *Gfap*-tk mice was reduced by approximately 90% (**Figure 1A**), imputing a critical role in tumor development to the enteric glia.

To confirm these findings using an alternative depletion model, we crossed *Gfap*-Cre mice with inducible diphtheria toxin receptor (iDTR) mice to render GFAP^+^ enteric glia sensitive to diphtheria toxin (DT). To confine the depletion of GFAP^+^ cells to the colon, we injected DT directly into the mucosal wall of the distal colon with the aid of an endoscope. This local administration efficiently depleted GFAP^+^ enteric glia, but not β-III-tubulin^+^ enteric neurons, in the distal colon (**Figures S2A, B**). In this model, depletion was also selective for enteric glia, as pH2A.X staining was similarly localized to SOX10^+^ HuC/D^-^ cells (**Figure S2C**), and total levels of β-III-tubulin in the distal colons of enteric-glia-depleted mice and non-depleted control mice demonstrated no significant differences (**Figure S2D**). We then induced CRC in non-depleted control mice and enteric-glia-depleted *Gfap*-Cre iDTR mice and assessed tumor burden. Depletion of GFAP^+^ enteric glia in *Gfap*-Cre iDTR mice resulted in a markedly reduced tumor burden compared to controls (**Figure 1B**), recapitulating the results observed in the *Gfap*-tk mice. To corroborate these findings with an alternative enteric-glial driver, we bred *Plp1*-CreER^T^ iDTR mice and induced CRC with AOM/DSS. When we depleted the enteric glia intracolonically using DT, we, again, observed reduced tumor burdens in the mice depleted of enteric glia compared to non-depleted controls (**Figure S2E**). Altogether, these findings demonstrate that the enteric glia function to promote tumor development in CRC.

### CD8^+^ T Cells, NK Cells, and Total Lymphocytes Do Not Contribute to the Effects of Enteric Glia Depletion on Tumor Burden

**Figure 2:**
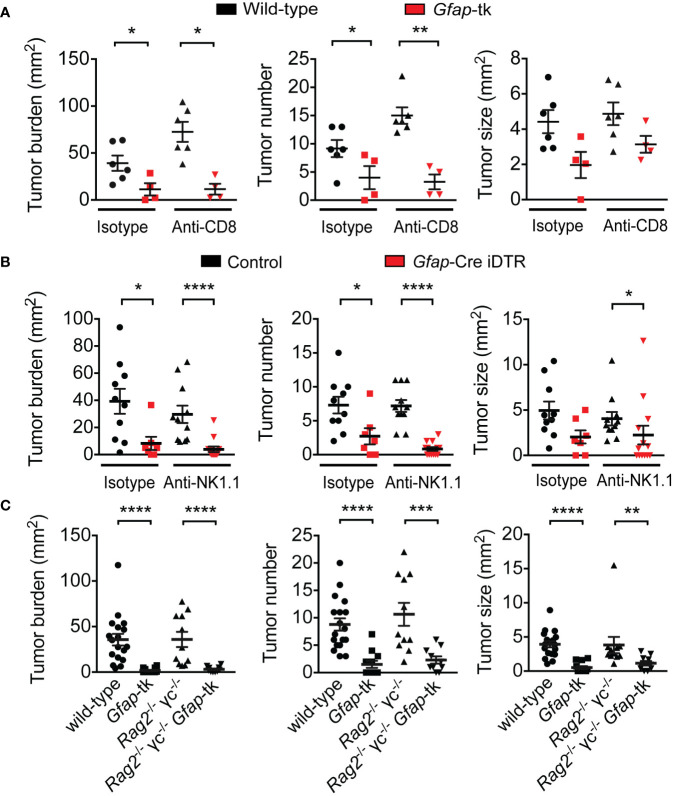
Reduction in tumor burden following enteric glia depletion does not depend on CD8^+^ T cells, NK cells, or total lymphocytes. **(A)** Analysis of tumors in wild-type and *Gfap*-tk AOM/DSS mice treated with ganciclovir and isotype or anti-CD8α depleting antibody for nine weeks following induction (n = 4–6 mice per group). **(B)** Analysis of tumors in non-depleted control AOM/DSS mice and enteric-glia-depleted *Gfap*-Cre iDTR AOM/DSS mice treated with isotype or anti-NK1.1 depleting antibody starting from induction (n = 7–13 mice per group). **(C)** Analysis of tumors in wild-type, *Gfap*-tk, *Rag2*^-/-^ γc^-/-^, and *Rag2*^-/-^ γc^-/-^ *Gfap*-tk AOM/DSS mice treated with ganciclovir starting from induction (n = 10–18 mice per group; data pooled from two independent experiments). Data are presented as mean ± SEM. *P* < 0.05 = *; *P* < 0.01 = **; *P* < 0.001 = ***; *P* < 0.0001 = ****, Mann-Whitney U test.

CD8^+^ cytotoxic T cells and NK cells are potent anti-tumor effector cells and are critical mediators of anti-tumor immunity (31–34). To examine whether the GFAP^+^ enteric glia could be promoting tumor development through suppression of CD8^+^ T cell or NK cell activity, we depleted CD8^+^ T cells (**Figure S3A**) or NK cells (**Figure S3B**) from enteric-glia-depleted AOM/DSS mice, using anti-CD8α and anti-NK1.1 antibodies, respectively, to determine whether the beneficial effect of enteric glia depletion was dependent on these cells. We discovered that this was not the case, as anti-CD8α-treated and anti-NK1.1-treated enteric-glia-depleted mice exhibited reduced tumor burdens similar to that observed in the isotype-treated controls (**Figures 2A, B**).

To determine whether lymphocytes as a whole were required for the protective effect of enteric glia depletion, we bred *Rag2*^-/-^ γc^-/-^ mice to *Gfap*-tk mice to obtain *Gfap*-tk mice lacking all lymphocyte populations, then induced CRC in the mice with AOM/DSS and depleted the GFAP^+^ enteric glia with ganciclovir. Similar to the results observed with the CD8^+^ T cell and NK cell depletions, the absence of all lymphocyte populations did not impact the tumor burden reduction observed following depletion of enteric glia (**Figure 2C**). Overall, these data demonstrate that the mechanism responsible for the beneficial effect of enteric glia depletion is not dependent on lymphocytes.

### Enteric Glia Depletion Does Not Affect Colitis Development

**Figure 3:**
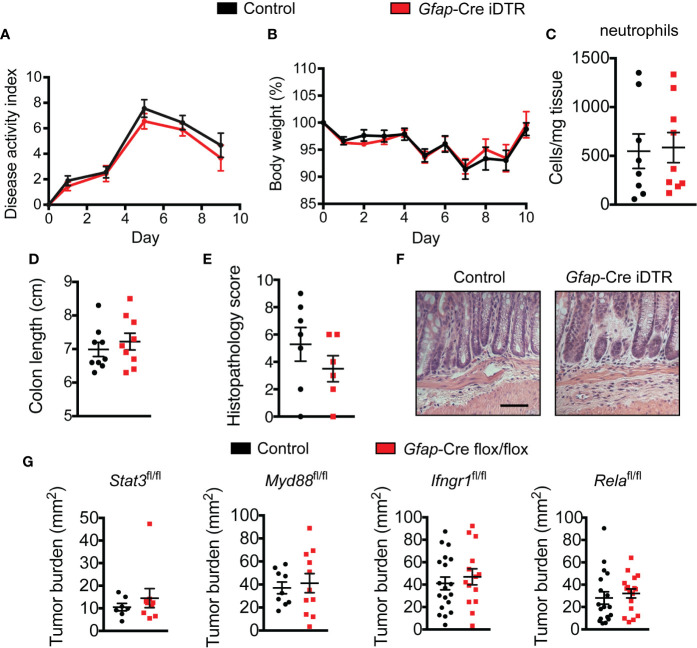
Enteric glia depletion does not affect colitis severity. **(A–E)** Disease activity index scores **(A)**, body weights **(B)**, neutrophil absolute counts in the distal colon **(C)**, colon lengths **(D)**, histopathology scores **(E)**, and hematoxylin and eosin images of the distal colon **(F)** for non-depleted control mice and enteric-glia-depleted *Gfap*-Cre iDTR mice undergoing acute DSS-induced colitis [n = 8–9 mice per group **(A–D)**; n = 6–7 mice per group **(E, F)**; representative of three experiments **(A, B, D)**]. Scale bar = 50 μm. **(G)** Analysis of tumors in various *Gfap*-Cre conditional knockout mice induced with AOM/DSS (n = 7–9 mice per group (*Stat3*^fl/fl^); n = 9–11 mice per group (*Myd88*^fl/fl^); n = 14–19 mice per group (*Ifngr1*^fl/fl^); n = 17–18 mice per group (*Rela*^fl/fl^); representative of three independent experiments (*Myd88*^fl/fl^); pooled from two independent experiments (*Ifngr1*^fl/fl^); pooled from three independent experiments (*Rela*^fl/fl^)). Data are presented as mean ± SEM.

As gut inflammation is known to accelerate tumor growth in the AOM/DSS model, and enteric glia can secrete and respond to inflammatory mediators (17, 35–37), we investigated whether the GFAP^+^ enteric glia could be promoting tumor development by exacerbating intestinal inflammation. To this end, we utilized DSS to induce acute colitis in enteric-glia-depleted mice and non-depleted control mice and assessed colitis severity. The disease activity index (**Figure 3A**), body weight loss (**Figure 3B**), neutrophil tissue infiltration (**Figure 3C**), colon length (**Figure 3D**), and histopathology score (**Figures 3E, F**) of the enteric-glia-depleted mice were all unchanged compared to control mice, indicating that GFAP^+^ enteric glia do not contribute significantly to the development of DSS-induced colitis.

We next considered the possibility that signaling through inflammatory pathways might contribute to the tumor-promoting effect of the GFAP^+^ enteric glia, as enteric glia can respond to inflammatory molecules such as lipopolysaccharide and interleukin-6 (17, 19, 35, 36) and can demonstrate signaling in these pathways following DSS treatment. To examine this possibility, we bred multiple conditional knockout mice to ablate these pathways selectively in the GFAP^+^ enteric glia: *Gfap*-Cre *Stat3*^fl/fl^, *Gfap*-Cre *Myd88*^fl/fl^, *Gfap*-Cre *Rela*^fl/fl^, and *Gfap*-Cre *Ifngr1*^fl/fl^ mice. Following CRC induction with AOM/DSS, mice with disrupted signaling in these pathways exhibited no difference in tumor burden when compared to non-Cre flox/flox littermate controls (**Figure 3G**). These data demonstrate that the aforementioned inflammatory pathways are not required for the pro-tumorigenic function of the GFAP^+^ enteric glia.

### Tumors in Enteric-Glia-Depleted Mice Exhibit Similar Properties Compared to Control Tumors

**Figure 4:**
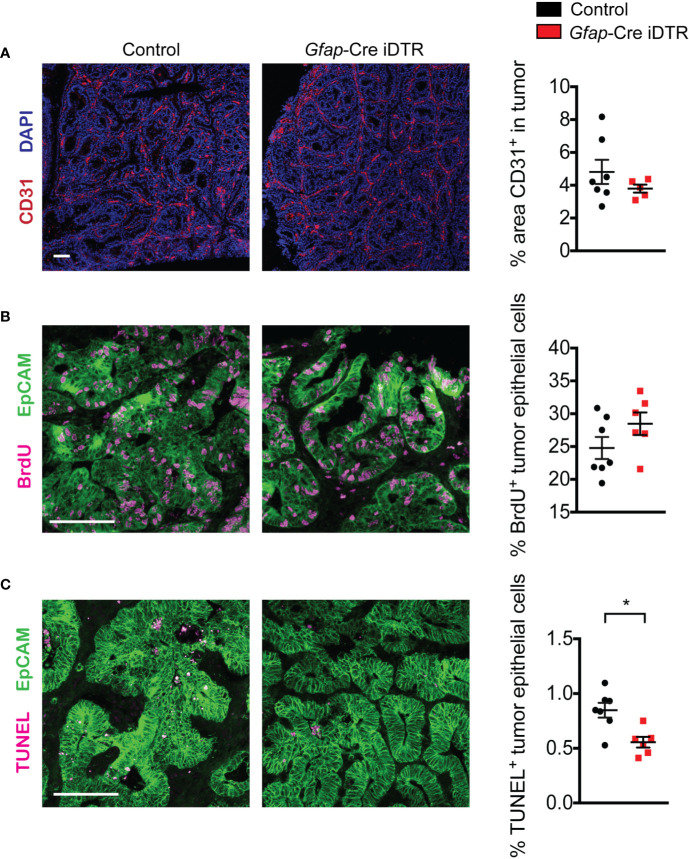
Tumors from enteric-glia-depleted mice and non-depleted control mice exhibit similar properties. **(A)** Representative immunofluorescence images of CD31 (red) and DAPI (blue) in tumors from non-depleted control AOM/DSS mice and enteric-glia-depleted *Gfap*-Cre iDTR AOM/DSS mice (n = 5–7 mice per group). Quantification is shown on the right. **(B, C)** Representative immunofluorescence images of EpCAM (green) and BrdU (magenta) **(B)** or TUNEL (magenta) **(C)** in tumors from non-depleted control AOM/DSS mice and enteric-glia-depleted *Gfap*-Cre iDTR AOM/DSS mice (n = 6–7 mice per group). Quantification is shown on the right. Scale bars are 100 μm. Data are presented as mean ± SEM. *P* < 0.05 = *, Mann-Whitney U test.

Next, we asked whether GFAP^+^ enteric glia depletion could be impacting tumor development through effects on tumor features such as angiogenesis or tumor cell proliferation and death. Based on CD31 staining, microvessel density did not differ between tumors that developed in enteric-glia-depleted mice and those that developed in non-depleted control mice (**Figure 4A**). Additionally, bromodeoxyuridine (BrdU) incorporation indicated that tumor cell proliferation was also similar between the two groups (**Figure 4B**). Terminal deoxynucleotidyl transferase dUTP nick end labeling (TUNEL) staining, however, revealed reduced tumor cell death in enteric-glia-depleted mice compared to non-depleted control mice (**Figure 4C**). Nevertheless, this reduction in cell death does not explain the reduced tumor burden observed following enteric glia depletion. Altogether, these data indicate that the absence of GFAP^+^ enteric glia has a limited impact on the properties of established tumors, suggesting that the GFAP^+^ glial cells may exert their pro-tumorigenic function early in tumor development.

### GFAP^+^ Enteric Glia Play a Critical Role in Early Tumor Development

**Figure 5:**
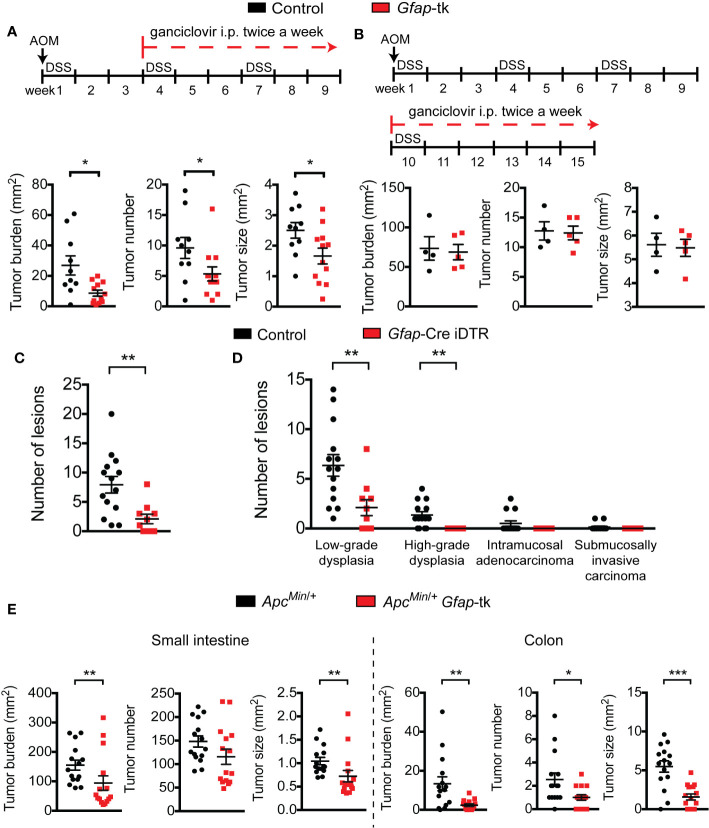
Enteric glia are important during early tumor development. **(A, B)** Analysis of tumors in wild-type and *Gfap*-tk AOM/DSS mice treated with ganciclovir starting from week four (n = 10–12 mice per group; pooled from two independent experiments) **(A)** or starting from week ten (n = 4–5 mice per group; representative of two independent experiments) **(B)**. **(C, D)** Total number of dysplastic lesions **(C)** and the number of lesions at each stage of tumor development **(D)** in the distal colon of non-depleted control AOM/DSS mice and enteric-glia-depleted *Gfap*-Cre iDTR AOM/DSS mice (n = 10–14 mice per group). **(E)** Analysis of tumors in the small intestines (left) and colons (right) of *Apc^Min^*^/+^ and *Apc^Min^*^/+^ *Gfap*-tk mice treated with ganciclovir (n = 15 mice per group; pooled from three independent experiments). Data are presented as mean ± SEM. *P* < 0.05 = *; *P* < 0.01 = **; *P* < 0.001 = ***; Mann-Whitney U test.

To determine at what stage of tumorigenesis the GFAP^+^ enteric glia might be exerting their pro-tumorigenic effect, we depleted these cells at different points during tumor development: starting at week four, when dysplastic lesions were evident, or starting at week ten, after tumors were established. Whereas depletion of the enteric glia starting from week four was sufficient to reduce tumor burden (**Figure 5A**), depletion starting at week ten had no effect (**Figure 5B**). This suggests that the enteric glia function to promote tumorigenesis early in tumor development but are not required for later growth of malignant lesions. Consistent with this idea, enteric glia in AOM/DSS mice were largely absent from the tumor mass, but were still present in the surrounding tissue (**Figure S4A**). This localization pattern was recapitulated in colonic sections from CRC patients (**Figure S4B**), using S100 calcium binding protein B (S100β) as a marker for enteric glia.

To further investigate the role of GFAP^+^ enteric glia in early tumor development, we examined the number and grade of precancerous dysplastic lesions present in the colons of non-depleted control mice and enteric-glia-depleted mice six weeks after AOM/DSS induction. At this time point, dysplastic lesions were evident, but overt cancers were not yet abundant. Assessment of the dysplastic lesions revealed that the total number of lesions was significantly reduced in enteric-glia-depleted mice compared to non-depleted control mice (**Figure 5C**). Additionally, enteric-glia-depleted mice exhibited a reduced number of more advanced neoplastic lesions (i.e. high-grade dysplasia and intramucosal or submucosally invasive adenocarcinoma), suggesting attenuated progression along the dysplasia-carcinoma sequence (**Figure 5D**, **Figure S4D**).

To confirm that precancerous tumor development is perturbed in the absence of enteric glia, we utilized the *Apc^Min^*^/+^ mouse model of familial adenomatous polyposis, as tumors in this model rarely progress past the premalignant adenoma stage. As observed with human CRC and the AOM/DSS model, enteric glia in *Apc^Min^*^/+^ mice were also reduced in the tumors (**Figure S4C**). We then crossed *Apc^Min^*^/+^ mice and *Gfap*-tk mice and treated the offspring with ganciclovir to deplete the enteric glia (**Figure S4E**). This resulted in reduced tumor burdens in the small intestines and colons of enteric-glia-depleted *Apc^Min^*^/+^ mice compared to the non-depleted controls (**Figure 5E**), supporting the concept that enteric glia play an important role during early tumor development. Altogether, our findings in the AOM/DSS and *Apc^Min^*^/+^ mice indicate that GFAP^+^ enteric glia contribute to CRC by promoting the early stages of tumorigenesis.

## Discussion

Despite the emerging evidence that enteric glia have numerous functions beyond local neuron support, their impact on the development of CRC has not been well investigated. Evidence of their contribution to CRC using *in vivo* tumor models has been especially lacking. In this study, we utilized the AOM/DSS and Apc^Min/+^ mouse models of intestinal tumorigenesis and two different methods of enteric glia depletion in order to examine their potential role in CRC.

One depletion method employed the use of *Gfap*-tk transgenic mice, in which actively replicating GFAP^+^ cells are rendered sensitive to ganciclovir-mediated cell death. This requirement for active replication is critical for limiting the extent of cell depletion, as GFAP^+^ cells in most major organs are quiescent and, therefore, do not become depleted (11). Previous use of the *Gfap*-tk model has been complicated by significant morbidities induced by the ganciclovir regimen. *Gfap*-tk mice receiving a continual dose of 100 mg/kg/day ganciclovir manifest severe small intestinal hemorrhaging and epithelial inflammation and damage within three weeks of ganciclovir administration and quickly succumb to the complications (11). Importantly, in our studies, the *Gfap*-tk mice did not experience any detectable small intestinal hemorrhaging and were able to survive the entire AOM/DSS regimen (around nine weeks) while undergoing continuous ganciclovir treatment. We believe this reduction in morbidity is a result of our milder ganciclovir regimen, which appears to have minimized the off-target effects and damage. As shown in **Figure S1**, we were able to achieve depletion of GFAP^+^ enteric glia in the AOM/DSS *Gfap*-tk mice without inducing cell death in neighboring neurons or in the small intestinal epithelium. Avoidance of the intestinal hemorrhaging with a low-dose ganciclovir regimen and the selectivity of depletion for GFAP-expressing cells are also described and discussed in other studies (38, 39).

Nonetheless, to avoid the “bystander effect” of the tk system altogether and to address concerns regarding the potential loss of neural progenitors in the mouse forebrain (39), we employed an alternative depletion method to corroborate our findings. To this end, we utilized the iDTR system, which does not require active cycling of the cells to achieve depletion. Thus, to minimize systemic toxicity, depletion of GFAP^+^ cells was confined to the distal colon using endoscopy-guided injections of DT. Importantly, depletion of GFAP^+^ cells in this model also appeared to be selective for enteric glia, as cell death was localized to SOX10^+^ HuC/D^-^ cells and the neuronal network appeared intact following DT injection. The concordance of the results in these two distinct depletion systems gives us confidence that the observed reduction in tumor burden is a consequence of depletion of GFAP^+^ enteric glia and not a result of off-target effects.

In a previous report (5), PLP1 was observed to be a more robust marker for enteric glia than GFAP, with the GFAP^+^ subset of enteric glia comprising only 40% of total PLP1-expressing cells. However, we found that GFAP expression is more widespread in the enteric glia population; for example, in contrast to the aforementioned findings, the extra-ganglionic glia in the circular muscle layer robustly express GFAP (see **Figure S1A** and **S2A**). Indeed, we observed GFAP^+^ enteric glia populations throughout all the colonic intestinal layers. This discrepancy may be a result of differences in anti-GFAP antibodies from different vendors, as we have experienced variable staining with different commercial products. Given these observations, we believe that the proportion of the GFAP^+^ subset of enteric glia is underestimated in Rao et al. Regardless, our findings of the pro-tumorigenic function of the glia were reproduced in *Plp1*-CreER^T^ iDTR mice. Of note, we observed a reduced effect size using the *Plp1*-CreER^T^ system, but we surmise this may be due to the additional step of requiring tamoxifen to induce Cre recombinase activity, which can lead to variable depletion efficiencies following DT injection.

Overall, our enteric glia depletion models indicate that GFAP^+^ enteric glia play a critical tumor-promoting role in the development of CRC, as depletion of the enteric glia dramatically reduced tumor burden in AOM/DSS mice. These cells, however, do not appear to play a requisite role throughout CRC progression, as depletion of GFAP^+^ glia after malignant tumors have already become established had no effect on the size or number of these tumors. Additionally, tumors that grew out of enteric-glia-depleted mice exhibited similar properties (i.e. vascularization and tumor cell proliferation and death) compared to tumors in non-depleted control mice, suggesting that the presence or absence of enteric glia does not greatly influence established malignant lesions. These findings are consistent with the observation by us and others (13, 22, 25) that enteric glia can be found adjacent to tumor epithelial cells but are largely absent from the tumor itself. The reason for this localization in the tumor mass is not known, but it suggests that continual direct contact with enteric glia is not obligatory for further tumor growth. Altogether, our findings suggest that GFAP^+^ enteric glia primarily play an important role in the early stages of CRC—a hypothesis supported by our data demonstrating reductions in dysplastic lesions in the AOM/DSS model and precancerous adenomas in the *Apc^Min^*^/+^ model following enteric glia depletion.

Interestingly, enteric glia depletion was found to be effective at reducing tumor burden in both the AOM/DSS model and the *Apc^Min^*^/+^ model, which represent two distinct settings of intestinal tumor formation. Whereas the tumors that develop in the AOM/DSS model are driven by random mutagenesis and colitis-dependent acceleration of malignant transformation, tumors in the *Apc^Min^*^/+^ model are initiated by the disruption of a single gene and develop in the absence of a chronic inflammatory background, a setting more reminiscent of sporadic CRC development. These data suggest that the pro-tumorigenic role of enteric glia may be conserved among CRC of different etiologies. Importantly, given that GFAP is expressed in a subset of ileal epithelial cells (30), the reduced tumor burden observed in the *Gfap*-tk *Apc^Min^*^/+^ mice could be attributed to direct killing of GFAP^+^ tumor epithelial cells rather than to the loss of enteric glia. However, using *Gfap*-Cre LSL-tdTomato reporter mice to fate map GFAP^+^ cells, we found that these GFAP^+^ intestinal epithelial cells are located only in the small intestine and not the large intestine (data not shown). Lack of GFAP expression in the colonic epithelial cells is additionally supported by the observation that the hemorrhaging and damage that has been reported in *Gfap*-tk mice appears to be confined to the ileum and does not extend to the large intestine (11, 30). Thus, the reduction in colonic tumor burden in the *Gfap*-tk *Apc^Min^*^/+^ mice demonstrates that direct killing of epithelial cells cannot fully account for the decreased tumor burden in these mice, supporting a protumorigenic role for enteric glia in this model.

Our investigations into the mechanism responsible for the tumor reduction following enteric glia depletion largely focused on examining immune and inflammatory mechanisms known to influence tumor growth in AOM/DSS mice. Surprisingly, our findings ruled out a role for classical anti-tumor effector cells such as CD8^+^ T cells and NK cells, as mice depleted of either of these subsets still exhibited dramatic reductions in tumor burden following the loss of the GFAP^+^ glia. We also explored the possibility that enteric glia might be promoting tumor development by exacerbating DSS colitis. This mechanism seemed likely, as enteric glia are widely reported to secrete inflammatory factors and are implicated in modulating inflammation in the gut (19, 21, 35, 40). However, depletion of the GFAP^+^ enteric glia had no measurable effect on DSS-induced colitis severity. This finding agrees with one report (30) but contrasts with another (15). This discrepancy in the literature might be attributed to differences in study approach (i.e. a conditional knockout vs cell depletion), but ultimately, further studies are required to clarify the contribution of enteric glia to intestinal inflammation. Additionally, although enteric glia appear to be activated by intestinal inflammation, selectively knocking out a number of inflammatory response pathways (e.g. STAT3, MYD88, RELA, and IFNGR1) in GFAP^+^ enteric glia did not affect their pro-tumorigenic function, supporting the view that the role of GFAP^+^ enteric glia in CRC might be independent of inflammatory mechanisms. This is interesting, as a recent study reported that IL-1 signaling in enteric glia could promote colon cancer stem cell stemness and tumorgenicity through enhanced PGE2 secretion (25). However, in our model, loss of downstream IL-1 signaling mediators, such as MYD88 and RELA, did not affect the tumor-promoting function of the enteric glia. Thus, although our main finding regarding the protumorigenic role of enteric glia aligns with Valès et. al., the mechanisms and contexts may differ.

Overall, our results reveal new insights into the role of enteric glia in the pathogenesis of CRC. GFAP^+^ enteric glia promote tumorigenesis during early tumor development, but do so without inhibiting anti-tumor immunity or exacerbating intestinal inflammation. Although the responsible mechanism is yet undefined, the profound impact of enteric glia depletion on tumor development suggests that novel therapeutic strategies targeting these cells could prove useful for CRC prevention.

## Data Availability Statement

The raw data supporting the conclusions of this article will be made available by the authors, without undue reservation.

## Ethics Statement

The studies involving human participants were reviewed and approved by Stanford Institutional Review Board. Written informed consent for participation was not required for this study in accordance with the national legislation and the institutional requirements. The animal study was reviewed and approved by Stanford University Institutional Animal Care and Use Committee.

## Author Contributions

RY and NB conceived the study, performed all experiments, and wrote the manuscript. JK helped in designing the research. JS and MD provided pathology assistance. TP and SB provided technical assistance. AH provided patient specimens for histology. EE supervised the study and wrote the manuscript. All authors contributed to the article and approved the submitted version.

## Funding

This work was supported by NIH grants R01 CA196657 and U54 CA209971. TP is supported by NIH NRSA F30CA196145. RY is supported by the Stanford Smith Fellowship. SB is supported by the Stanford School of Medicine Dean’s Fellowship.

## Conflict of Interest

The authors declare that the research was conducted in the absence of any commercial or financial relationships that could be construed as a potential conflict of interest.
