# Supplementary material for: Enteric Glia Play a Critical Role in Promoting the Development of Colorectal Cancer
Source: Front Oncol. 2020 Nov 13;10:595892. doi: 10.3389/fonc.2020.595892 (PMC7691584; doi:10.3389/fonc.2020.595892)
Supplement: Supplementary file 1 [file Table_1.docx]

**SUPPLEMENTAL FIGURES**

| 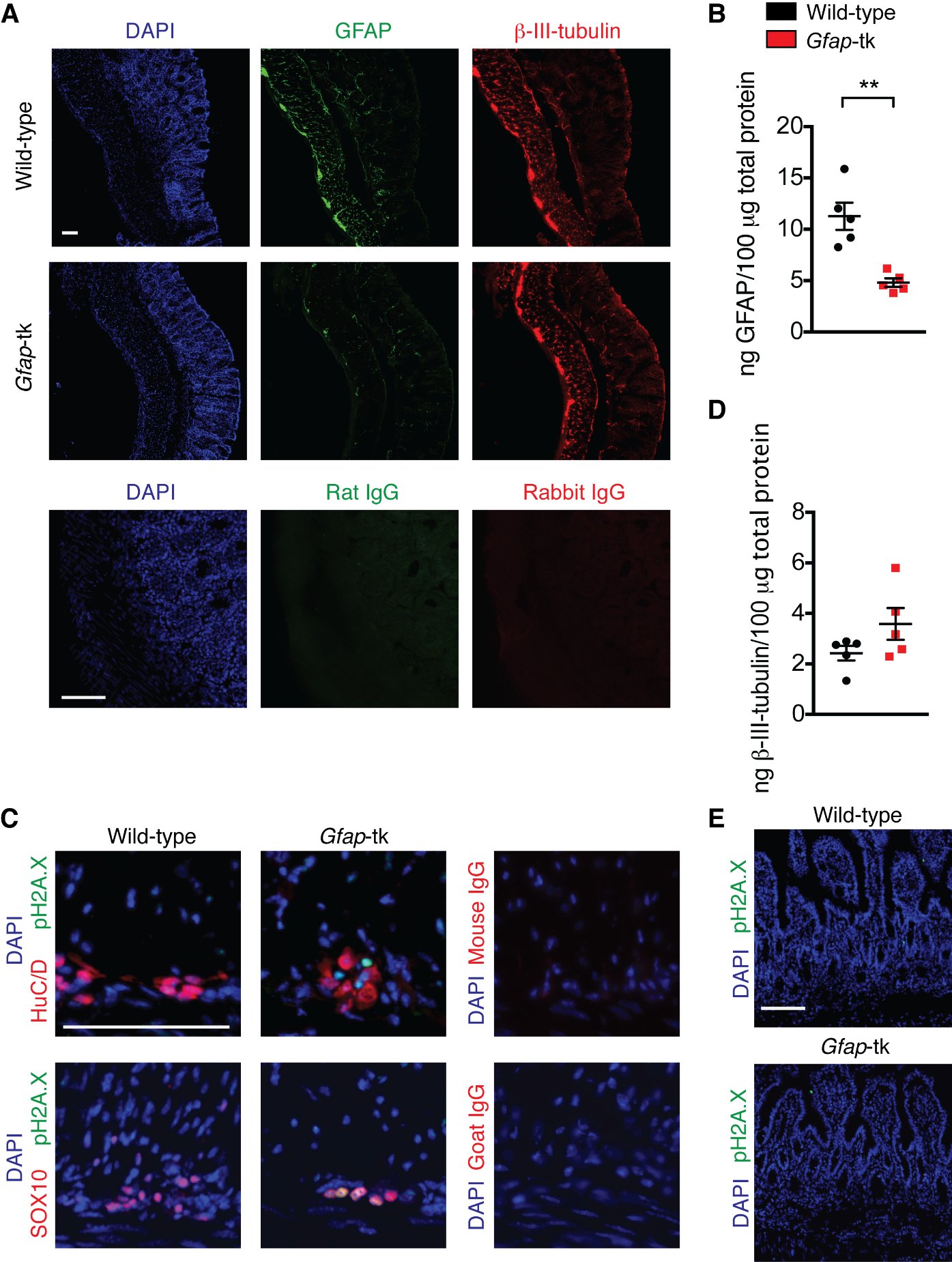 |
| --- |
| **Figure S1. Enteric glia depletion in *Gfap*-tk mice.** (A) Representative immunofluorescence images of DAPI (blue), GFAP (green), and β-III-tubulin (red) in colons from wild-type and *Gfap*-tk mice induced with AOM/DSS and treated with ganciclovir. Isotype controls are shown in the bottom panels. (B) GFAP ELISA of distal colonic lysates from wild-type and *Gfap*-tk mice induced with AOM/DSS and treated with ganciclovir (n = 5 mice per group). (C) Immunofluorescence images of DAPI (blue), pH2A.X (green), and SOX10 or HuC/D (red) in colons from wild-type and *Gfap*-tk mice induced with AOM/DSS and treated with ganciclovir. Isotype controls are shown in the panels to the right. (D) β-III-tubulin ELISA of distal colonic lysates from wild-type and *Gfap*-tk mice induced with AOM/DSS and treated with ganciclovir (n = 5 mice per group). (E) Immunofluorescence images of DAPI (blue) and pH2A.X (green) in small intestines from wild-type and *Gfap*-tk mice induced with AOM/DSS and treated with ganciclovir. Scale bars are 100 μm. Data are presented as mean±SEM. *P*<0.05 = *; *P*<0.01 = **; *P*<0.001 = ***; *P*<0.0001 = ****, Mann-Whitney U test. |
| **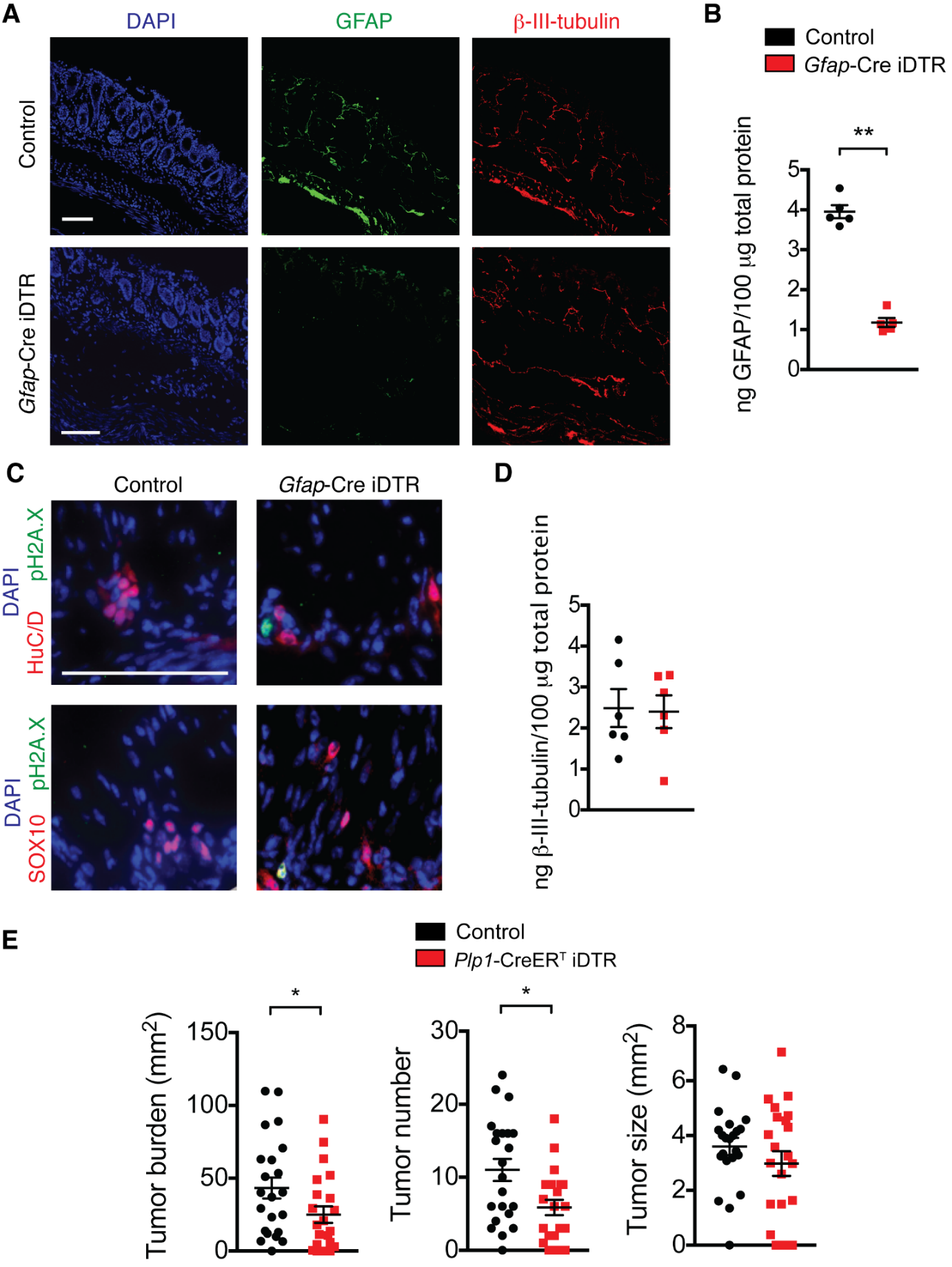** |
| **Figure S2. Enteric glia depletion in *Gfap*-Cre iDTR mice and tumor burden analyses in *Plp1*-CreER^T^ iDTR mice.** (A) Representative immunofluorescence images of DAPI (blue), GFAP (green), and β-III-tubulin (red) in colons from non-depleted control mice and enteric-glia-depleted *Gfap*-Cre iDTR mice. (B) GFAP ELISA of distal colonic lysates from non-depleted control AOM/DSS mice and enteric-glia-depleted *Gfap*-Cre iDTR AOM/DSS mice (n = 5 mice per group). (C) Immunofluorescence images of DAPI (blue), pH2A.X (green), and SOX10 or HuC/D (red) in colons from non-depleted control AOM/DSS mice and enteric-glia-depleted *Gfap*-Cre iDTR AOM/DSS mice. (D) β-III-tubulin ELISA of distal colonic lysates from non-depleted control AOM/DSS mice and enteric-glia-depleted *Gfap*-Cre iDTR AOM/DSS mice (n = 6 mice per group). (E) Analysis of tumors in non-depleted control AOM/DSS mice and enteric-glia-depleted *Plp1*-CreER^T^ iDTR AOM/DSS mice (n = 22 mice per group; pooled from two independent experiments). Scale bars are 100 μm. Data are presented as mean±SEM. *P*<0.05 = *; *P*<0.01 = **; *P*<0.001 = ***; *P*<0.0001 = ****, Mann-Whitney U test. |

| 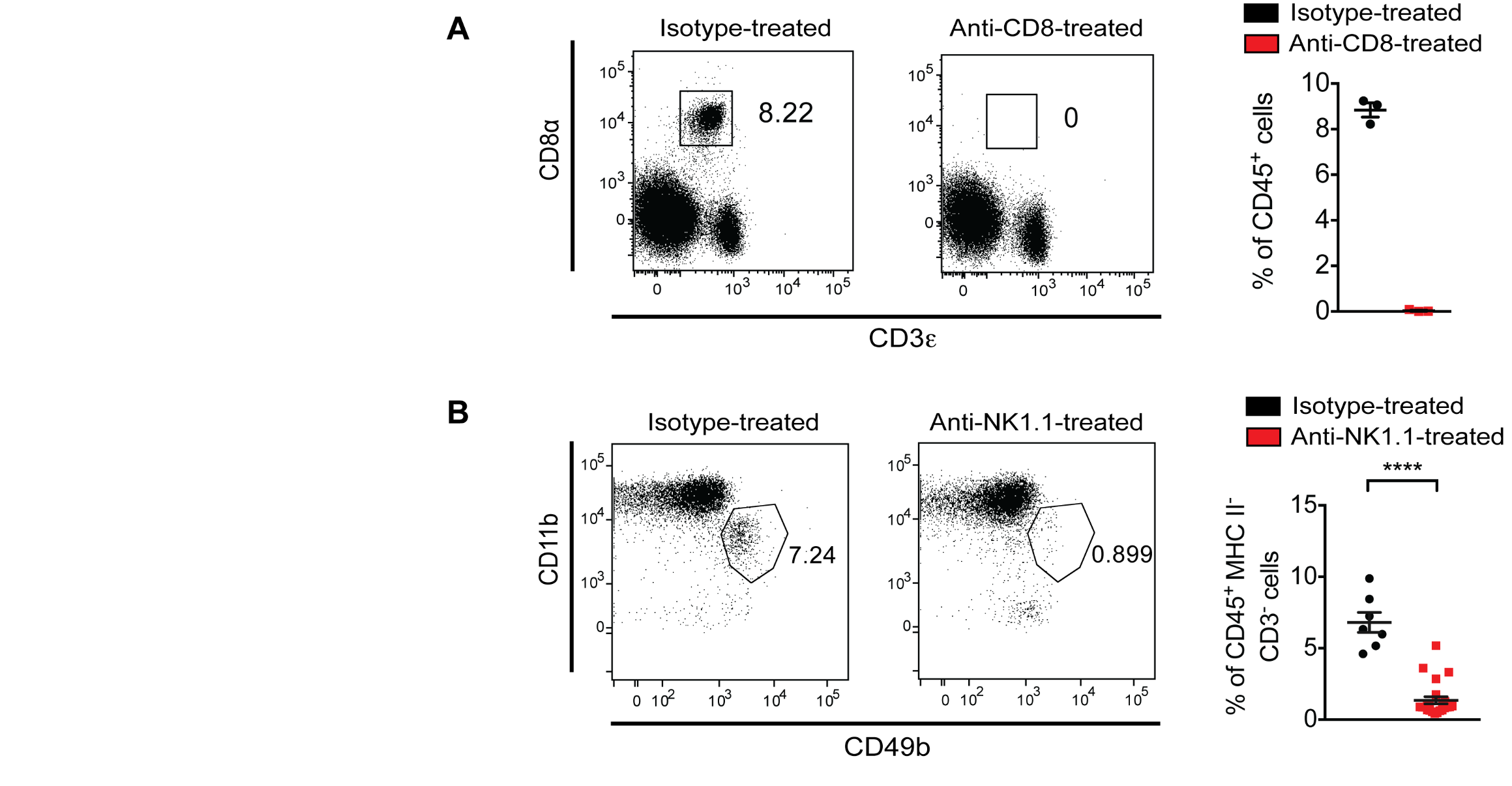 |
| --- |
| **Figure S3. Depletion of CD8^+^ T cells and NK cells in antibody-treated mice.** (A) Representative flow cytometry plots of cells from the blood of isotype-treated and anti-CD8α-treated mice (n = 3 mice per group). Quantification of percent of CD8^+^ T cells of total CD45^+^ cells is shown on the right. (B) Representative flow cytometry plots of cells from the blood of isotype-treated and anti-NK1.1-treated mice (n = 7-23 mice per group). Quantification of percent of NK cells of CD45^+^ MHC II^-^ CD3^-^ cells is shown on the right. Data are presented as mean±SEM. *P*<0.05 = *; *P*<0.01 = **; *P*<0.001 = ***; *P*<0.0001 = ****, Mann-Whitney U test. |

| 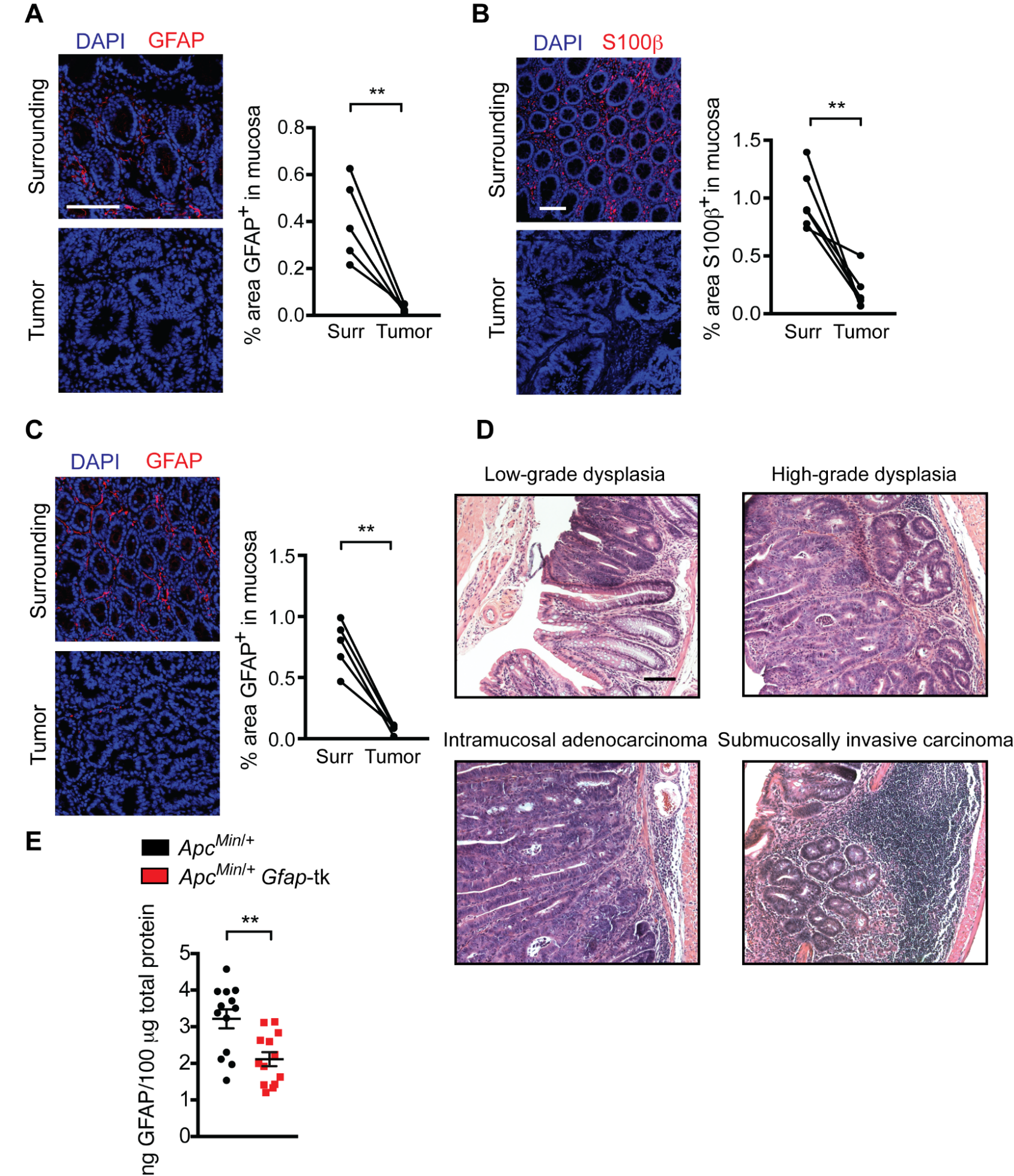 |
| --- |
| **Figure S4. Images of enteric glia in tumors and depletion of enteric glia in *Apc^Min^*^/+^ *Gfap*-tk mice.** (A-C) Representative immunofluorescence images of S100β (red) and DAPI (blue) in surrounding tissue (top) and matched tumor tissue (bottom) from CRC patients (n = 6 patients) (B) and of GFAP (red) and DAPI (blue) in surrounding tissue (top) and tumor tissue (bottom) from AOM/DSS mice (n = 5 mice) (A) and *Apc^Min^*^/+^ mice (n = 5 mice) (C). Quantifications are shown on the right. (D) Representative hematoxylin and eosin images of neoplastic lesions in the distal colons of AOM/DSS mice. (E) GFAP ELISA of small intestinal lysates from *Apc^Min^*^/+^ and *Apc^Min^*^/+^ *Gfap*-tk mice treated with ganciclovir (n = 13 mice per group). Scale bars are 100 μm. Data are presented as mean±SEM. *P*<0.05 = *; *P*<0.01 = **; *P*<0.001 = ***; *P*<0.0001 = ****, paired *t*-test (A-C), Mann-Whitney U test (E). |
